# The Association between Blood Pressure Trajectories and Risk of Cardiovascular Diseases among Non-Hypertensive Chinese Population: A Population-Based Cohort Study

**DOI:** 10.3390/ijerph18062909

**Published:** 2021-03-12

**Authors:** Fang Li, Qian Lin, Mingshu Li, Lizhang Chen, Yingjun Li

**Affiliations:** 1 Department of Epidemiology and Health Statistics, Xiangya School of Public Health, Central South University, Changsha 410078, China; lifang_csu@csu.edu.cn; 2 Hunan Provincial Key Laboratory of Clinical Epidemiology, Changsha 410078, China; 3 Department of Nutrition Science and Food Hygiene, Xiangya School of Public Health, Central South University, Changsha 410078, China; linqian@csu.edu.cn (Q.L.); limingshu@csu.edu.cn (M.L.); 4 Department of Epidemiology and Health Statistics, School of Public Health, Hangzhou Medical College, Hangzhou 310053, China

**Keywords:** blood pressure trajectories, cardiovascular diseases, cohort study, growth mixture modeling, primary prevention

## Abstract

Although previous studies have discussed the association between trajectories of blood pressure (BP) and risk of cardiovascular diseases (CVDs), the association among the non-hypertensive general population of youth and middle age has not been elucidated. We used the growth mixture model to explore the trajectories of BP among the non-hypertensive Chinese population and applied Cox regression to evaluate the association between trajectories of BP and the risk of stroke or myocardial infarction (MI). Systolic blood pressure (SBP) and diastolic blood pressure (DBP) were categorized into three classes, respectively. Statistically significant associations were observed between SBP trajectories and stroke (range of adjusted hazard ratios (aHR): 1.369–3.837) or MI (rang of aHR = 6.047–13.017). Association between DBP trajectories and stroke (aHR: 3.685) or MI (range of aHR = 1.312–2.821) were also observed, although they did not reach statistical significance. Trajectories of SBP were more important risk factors than that of DBP in stroke and MI in our study population. BP management is important among pre-hypertensive adults to prevent stroke and MI when they age. Well-designed research with a larger sample size is required to confirm our findings and develop efficient methods to prevent CVDs.

## 1. Introduction

Stroke and myocardial infarction (MI) are common cardiovascular diseases (CVDs). Stroke is a group of diseases characterized by ischemia or hemorrhage of the cerebral and MI is a common situation of ischemic heart disease [1]. Both of the two diseases are medical emergencies and leading causes of death and disability, and the economic costs for treatment and rehabilitation are substantial [2]. During the past decades, the global burden of diseases showed an epidemiological shift. Morbidity and mortality due to non-communicable diseases such as cardiovascular and cerebrovascular diseases are increasing while morbidity and mortality because of communicable diseases such as tuberculosis are declining [3]. In 2019, ischemic heart disease (including MI) and stroke ranked in the top three leading causes in all ages globally and consist of 7.2% and 5.7% of disability-adjusted life-years (DALYs), respectively [4]. China experiences the same pattern. In 2017, ischemic heart disease and stroke ranked as the top causes of death and years of life lost (YLLs) [5]. From 1990 to 2017, the percentage change in mortality of ischemic heart disease and stroke increased 41% and 155.4%, respectively [5]. In sum, stroke and MI are major public health problems; approaches focused on modifiable risk factors to prevent stroke and MI are essential to control this serious situation.

Prehypertension, characterized by gently evaluated blood pressure (BP) in the range of 120–139/80–89 mmHg, is highly prevalent worldwide. Globally, the estimation of prehypertension ranged from 20% to 50% [6,7,8,9,10]. In China, the prevalence of prehypertension increased in recent years, with the estimation around 20% to 40% [6,11,12,13]. Prehypertension can progress to hypertension [14,15], and it associates with increased risks of various diseases and disorders, such as impairment of cognitive function, kidney disease, metabolic syndrome, coronary heart disease, stroke, and MI [13,15,16,17,18,19,20,21]. Although prehypertension is the risk factor of a wide range of diseases, it is reversible and the adverse outcomes are preventable. For example, epidemiological evidence showed that every 10 mmHg reduction in systolic blood pressure (SBP) would help to reduce the risk of stroke by 41% (33–48%) and coronary heart disease risk by 22% (17–27%) [22]. Thus, in the perspective of disease prevention, issues that relate to prehypertension management call for more attention.

In recent years, the studies on the association between the longitudinal change of BP (i.e., trajectories of BP) and stroke or MI have been gaining popularity. However, the vast majority of studies focused on the BP trajectories among CVDs patients; only a small proportion of studies discussed the BP trajectories in general populations [23,24,25,26,27]. Furthermore, it was rare that the studies reported the association between trajectories of BP and stroke or MI among the non-hypertensive Chinese population. By measurements of BP at the beginning and the end of observation, Fan, J. H. and colleagues studied the longitudinal change in BP in a Chinese county [28]. However, this practice may lead to a loss of substantial information during the observational period, resulting in the possibility of misclassification of BP trajectories [28]. Xu, Y. and colleagues conducted a study using latent class growth modeling (LCGM) to identify BP trajectories [29]. Nonetheless, they only assessed the association between trajectories of SBP and CVDs, the association between diastolic blood pressure (DBP) and CVDs was left out [29]. Thus, a comprehensive estimation of BP trajectories and the association with CVDs is lacking in the Chinese population.

Based on these backgrounds, we conducted this study aimed to (1) explore the trajectories of BP among the non-hypertensive Chinese population; and (2) evaluate the association between trajectories of BP and the risk of stroke or MI.

## 2. Materials and Methods

### 2.1. Study Design and Population

This study was based on the national data from the China Health and Nutrition Survey (CHNS), a population-based longitudinal project collaboratively conducted by the Carolina Population Center at the University of North Carolina at Chapel Hill and the National Institute for Nutrition and Health (NINH, former National Institute of Nutrition and Food Safety) at the Chinese Center for Disease Control and Prevention (CCDC). The details of the CHNS are described on the website of the project [30]. This ongoing cohort was initiated in 1989 and followed-up for every 2 to 4 years to examine the effects of the health, nutrition, and family planning policies and programs implemented by national and local governments and to see how the social and economic transformation of Chinese society is affecting the health and nutritional status of Chinese population. Until 2015, 42,829 individuals from 11,130 households participated by a multistage, random cluster sampling process from 15 provinces and autonomous cities/districts.

**Figure 1:**
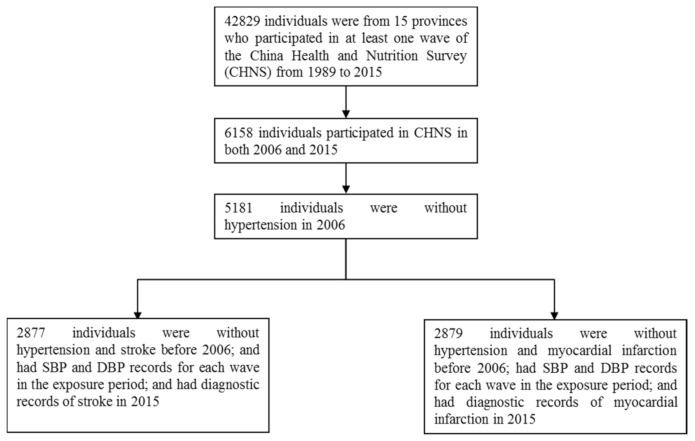
The flowchart of enrollment.

For our study, data of the CHNS from four waves (2000, 2004, 2006, and 2015) were retrieved to establish two individual datasets, one for stroke analysis and another for MI analysis. The datasets were divided into two periods, the exposure period (wave 2000, 2004, and 2006) and the outcome period (wave 2006 to 2015). The participants enrolled for the dataset of stroke were the ones who (1) had SBP and DBP records for each wave in the exposure period; (2) were not diagnosed with hypertension and stroke before 2006; and (3) had diagnostic records of stroke in 2015. The participants enrolled for the dataset of MI were the ones who (1) had SBP and DBP records for each wave in the exposure period; (2) were diagnosed without hypertension and MI before 2006; and (3) had diagnostic records of MI in 2015. Finally, a total of 2877 individuals were enrolled for stroke and 2879 individuals were enrolled for MI for classification. Figure 1 shows the flow chart of enrollment.

### 2.2. Ethical Approval

The study protocol of the CHNS was approved by the institutional review board from the University of North Carolina at Chapel Hill and the National Institute for Nutrition and Food Safety, China Centre for Disease Control and Prevention. Written informed consent was collected from all participants.

### 2.3. Data Collection

#### 2.3.1. Main Outcomes

BP was measured three times by a mercury sphygmomanometer on the right arm of each participant at each visit after a 10-min seated rest for the exposure period (2000, 2004, and 2006) [31]. We calculated the average of three measurements of SBP and DBP of each wave, respectively. Prehypertension was defined as BP of 120–139/80–89 mmHg among non-hypertensive individuals according to the 2018 Chinese guidelines for the management of hypertension [32].

Outcomes of interest were new-onset stroke and MI among non-hypertensive individuals, from 2006 to 2015. Participants gave self-reported information of hypertension, stroke, and MI status by answering the following questions: “Has the doctor ever given you the diagnosis of hypertension?”, “Has the doctor ever given you the diagnosis of stroke?” and “Has the doctor ever given you the diagnosis of myocardial infarction?” The positive answer was defined as the patient having the relevant diseases. We defined the onset time of stroke or MI by the answer of participants to the question “How old were you when you received the diagnosis of stroke?” and “How old were you when you received the diagnosis of myocardial infarction?” We calculated the survival time by the self-reported age of onset of stroke or MI, subtracting their age in 2006.

#### 2.3.2. Covariates

Covariates were social-demographic variables, lifestyle factors, body mass index (BMI) at the first three waves, energy intake in 2006, and activity level in 2006.

Social-demographic variables (age, location, ethnicity, sex, and highest educational level) and lifestyle factors (smoking and drinking status in 2006) were retrieved from self-reported questionnaires. Age was divided into four levels, younger than 40, 51–50 years, 51–60 years, and older than 61 years. BMI was calculated using measurements of height and weight by trained medical practitioners, then categorized into four levels, i.e., underweight (<18.5 kg/m^2^), normal (18.5–23.9 kg/m^2^), overweight (24.0–27.9 kg/m^2^), and obese (≥28 kg/m^2^) [33].

Dietary information was collected by trained field interviewers using the 24-h individual recall method on three consecutive days which were randomly allocated from Monday to Sunday. During the daily interview, individuals were asked to recall the food consumption and to report the types, amounts, type of meal, and place of consumption of all food items during the previous day, with the aid of food models and pictures. The dietary data were linked with a nutrient data bank for the new version of Chinese food composition tables and the averages of energy intake values were calculated [34].

Information of activity level was collected by self-reported answers to questions addressing physical activity involved in work and questions related to energy-expenditure, such as “How much time did you spend on the light/middle/heavy physical activities?” Examples of different activity levels were given to interviewees in order to help them better quantify the daily physical activities. Then these data were categorized into four levels, light, middle, heavy, and no working ability.

### 2.4. Statistical Analyses

If distributed normally, continuous variables were presented as means and standard deviations; otherwise, medians and interquartile ranges were applied. Categorical variables were presented by numbers and proportions. Continuous variables were compared using one-way ANOVA if distributed normally or Kruskal–Wallis tests if not. Categorical variables were using chi-square tests or Fisher’s exact tests, respectively. Missing values of continuous variables were estimated by the EM algorithm based on the maximum likelihood while the ones of categorical variables were processed by the multiple imputation method [35,36,37]. The analyses of associations between BP trajectories and stroke or MI were performed by the Cox regression model [24,38]. Multivariables were selected by the method of stepwise forward (likelihood ratio) to avoid multicollinearity. The assumption of proportionality was confirmed by Kaplan–Meier curves for categorical variables and by a Schoenfeld residuals plot for continuous variables, respectively [39]. Hazard ratios (HR) and the 95% confidence interval (CI) were applied to describe the associations in Cox regression models [40].

Using Mplus (v7.4, developed by Muthén and Muthén, Los Angeles, CA, USA), the growth mixture model (GMM) approach was applied to model BP trajectories over the exposure period (2000, 2004, and 2006) and to identify distinct subgroups following similar patterns. GMM is one of the most flexible clustering developed analyses in recent years and was applied to group individuals into an optimal number of classes or subgroups [41,42]. The gender, age, and BMI of each wave were adjusted when the GMM was performed. We first compared two- to four- or five-class GMM models to identify the best-fitting one by sample size-adjusted Bayesian Information Criterion (BIC) (aBIC) and entropy. A smaller number of aBIC indicated a better-fitting model [43], while a larger value of entropy represented a smaller likelihood of misclassification [43,44]. The adjusted Lo–Mendell–Rubin likelihood ratio test (aLMR-test) and bootstrapped likelihood ratio test (BLRT) were used to compare the n-class model versus the n−1 class model [45,46]. The significant *p*-value (*p* < 0.05) suggested that the n-class model was well improved over the n-1-class model.

Other Statistical analyses were conducted by IBM SPSS (v25.0) (IBM, Armonk, NY, USA). The significance level was *p* < 0.05 unless otherwise specifically mentioned.

### 2.5. Sensitivity Analyses

Sensitivity analyses were conducted to explore the robustness of our results to missing data, modeling decisions, and age-stratified subgroups. We repeated the analyses with individuals without missing covariates to test the robustness of our observed association between the 3-class models for BP trajectories and stroke and MI, respectively. In addition, we inspected models based on 1 fewer and 1 more trajectory than the best-fitting models chosen for main analyses. Thirdly, because of the wide range of age in our study (range: 69), we wanted to understand whether our identified trajectories were similar in different age groups. Thus, we repeated analyses in the two subgroups divided by age in 2006 (≤50 years old versus >50 years old).

## 3. Results

### 3.1. Descriptive Analysis

A total of 2877 individuals were enrolled in the analysis for stroke and 2879 individuals were enrolled for MI, with the age of 50 (17) in 2006. Then they were followed up for the incidence of stroke or MI, with average person-years of 8.97 ± 0.44 and 8.98 ± 0.24, respectively. During the observation period, the incidence of stroke was 1.5% (44/2877), and the incidence of MI was 1.4% (41/2879).

The characteristics of the participants in 2006 are summarized in Table 1.

### 3.2. GMM for Blood Pressure

Table S1 presents model fit indices for BP trajectories (SBP and DBP, respectively) in datasets of stroke or MI, respectively. To determine the best-fitting model, aBIC, entropy, aLMR and BLRT were assessed comprehensively. In GMM for stroke, both the value of aBIC for SBP and DBP trajectories first decreased and then increased; the three-class models presented the lowest value of aBIC (69078.724 for SBP, 62755.633 for DBP). When comparing the four-class models to the three-class models, no significant difference was observed by aLMR (*p =* 0.6711 for SPB, *p* = 0.4997 for DPB), which indicated that the four-class model was not improved compared to the three-class model. The *p*-value of aLMR-test showed a significant difference when three-class model was compared to the two-class model in DPB but not in SBP (*p* = 0.0032 for DPB, *p* = 0.00845 for SPB). This indicated that the three-class model for SBP was not improved compared to the two-class model, while the three-class model for DBP was improved compared to the two-class model. However, in the *p*-value of BLRT indicated that the three-class models were the best models. Taking the abovementioned into consideration, the three-class solutions were determined to be the best models. Notably, the entropy of three-class models was 0.641 for SBP and 0.666 for DBP, which indicated a potential problem of misclassification. So, we performed sensitivity analyses for other models; the results are shown in Table S3. The case of GMM for MI was similar (Table S1); the three-class models were determined to be the best models. The fit indices of best-fitting models are shown in Table S1 in bold.

**Figure 2:**
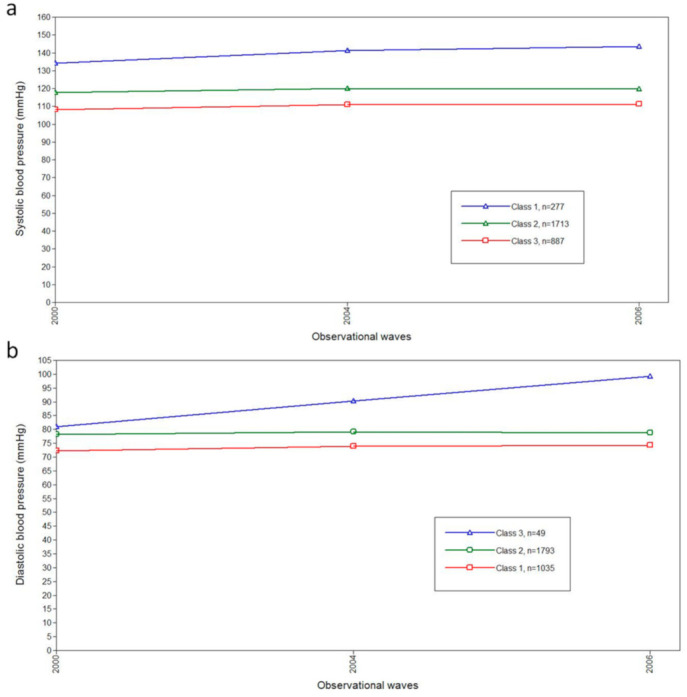
The blood pressure trajectories in the three-class model by GMM for stroke. (**a**) The plots for classifications of systolic blood pressure trajectories by GMM for stroke. (**b**) The plots for classifications of diastolic blood pressure trajectories by GMM for stroke. Abbreviations: mmHg, millimeter of mercury; GMM, growth mixture model.

**Figure 3:**
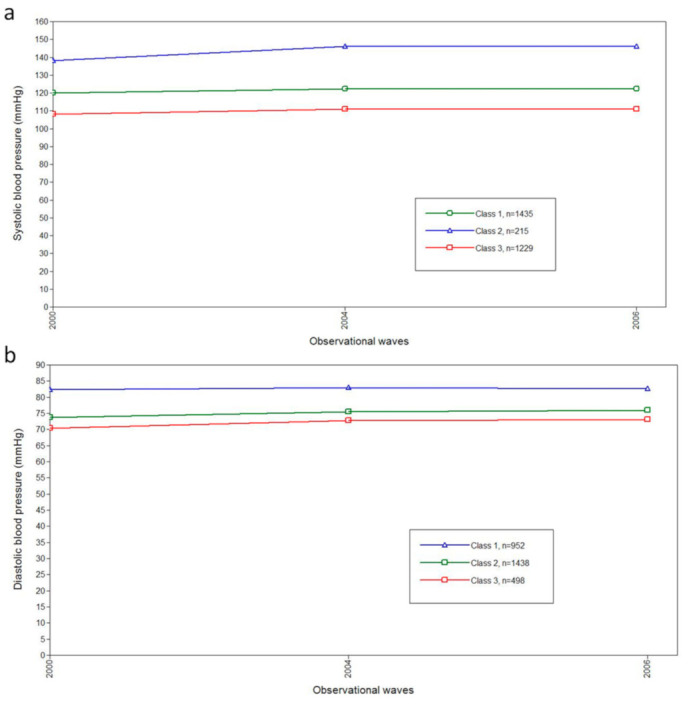
The blood pressure trajectories in the three-class model by GMM for myocardial infarction. (**a**) The plots for classifications of systolic blood pressure trajectories by GMM for myocardial infarction. (**b**) The plots for classifications of diastolic blood pressure by GMM for myocardial infarction. Abbreviations: mmHg, millimeter of mercury; GMM, growth mixture model.

Figure 2 and Figure 3 show three-class models determined by the analyses. To some degree, all participants showed an increasing pattern of BP during the observation period. As shown in Figure 2 and Figure 3, the classes with the most individuals were middle class in both SPB and DBP trajectories (Green lines). By contrast, the highest classes showed the fewest participants, with the means of BP starting in the intervals of prehypertension (120–139/80–89 mmHg) and gradually increasing. The classification probability and the class counts of each trajectory are presented in Table S1.

### 3.3. Characteristics across Trajectory Groups

Table 2 and Table 3 present the characteristics across BP trajectory groups (SBP and DBP) in the datasets of stroke and MI, respectively. For stroke, significant statistical differences were detected among SBP trajectory groups in age level, gender, education level, smoking in 2006, still smoking in 2006, drinking in 2006, BMI category in 2006, energy intake in 2006, and activity level in 2006 (all *p* < 0.05). Among DBP trajectory groups, statistically significant differences were observed among age levels, genders, education levels, smoking in 2006, still smoking in 2006, drinking in 2006, BMI category in 2006, and energy intake in 2006 (all *p* < 0.05). Notably, the number of individuals in Class 3 of DBP trajectory groups was limited (*n* = 49).

For the dataset of MI, it was similar to stroke, besides the distribution of two characters in DBP trajectory groups. Statistically significant differences were additionally observed in ethnicity and activity level in 2006 (all *p* < 0.05).

### 3.4. Cox Regression Analyses for Stroke and MI

Table 4 presents the associations between trajectories of SBP or DBP and stroke or MI, respectively. After adjustment for confounding variables presented in Table 2 and Table 3, significant differences in risks of stroke or MI were detected among BP trajectory groups (adjusted model 1 and adjusted model 2).

For stroke, after selection of variables by the forward method (likelihood ratio), the variable of SBP trajectory entered the function. Compared to Class 3, the range of adjusted HR (aHR) was 1.369 to 3.837. By contrast, the variable of DBP trajectory contributed poorly and did not enter the function.

In the estimation of the risk between SBP trajectories and MI, the aHR (95% CI) was 6.047 (2.121–17.239) for Class 1 and 13.017 (4.009–42.470) for Class 2 when compared to Class 3 (adjusted model 1). The relationship was also observed between DBP trajectories and MI, with the range of aHR (95% CI) 1.312 to 2.821. Adjusted model 2 which included trajectories of SPB and DPB simultaneously and selected variables by the forward method (likelihood ratio) indicated that the association between stroke and SBP was stronger than with DBP.

### 3.5. Sensitivity Analyses

Assessments of the influence of missing values are presented in Table S2, which shows the associations between trajectories of SBP or DBP and stroke or MI among participants without missing covariates in three-class models. Patterns of risk between BP trajectories and stroke or MI were similar to the main analyses: the high BP trajectories related to a higher risk of stroke and MI.

When compared to the chosen three-class models, no additional insights were provided by the two-class and four-class models. The trajectories were presented by Figure S1 to Figure S4 and the fit indices for BP trajectories were shown in Table S1. The Cox regression analyses indicated the association between BP trajectories and risk of stroke or MI in the two-class and four-class model (Table S3).

Another sensitivity analysis in which the trajectories were modeled based on the stratification of the age of 2006 (≤50 years old versus >50 years old) was carried out. Although the means of BP trajectories of the young subgroups were lower than the senior subgroups, trajectories were similar to the main results as well as the risk of stroke or MI (Figure S5 to Figure S8, Table S4).

## 4. Discussion

Although previous studies have discussed the association between the trajectories of BP and risk of CVDs, the association among the non-hypertensive general population has not been elucidated. To the best of our knowledge, this is the first study based on a national cohort that explored the trajectories of BP among the non-hypertensive Chinese general population and evaluated the associations between trajectories of BP and risk of stroke or MI. Our study found that the trajectories of SBP and DBP were categorized into three classes by GMM. Significant positive associations were detected for trajectories of BP and risk of stroke and MI, respectively. In addition, it was the increased trajectory of SBP rather than DBP that was the major risk factor of developing stroke or MI. These findings could help provide new insight into the prevention and management of CVDs in the general population.

Our findings indicated that the trajectories of SBP increased in all classes, which was in line with previous studies [47,48,49]. The study conducted by Wolf-Maier, K. and colleagues showed an age-related increase in SBP, with an estimation of 7 mmHg per decade in Westerners over the age of 40 [48]. As regards trajectories of DBP, previous studies reported a bell-shaped curve: it increased from middle age and declined in later life; the turning point was around 50 to 60 [48,49]. However, we found the trajectories of DBP increased, although not dramatically, in all three classes. A possible explanation for the discrepancy was the difference in age of participants. Participants were aged 35 to 74 in the study conducted by Wolf-Maier, K. and colleagues while individuals were aged 30 to 84 in the study conducted by Franklin, S. S. and colleagues [48,49]. By contrast, in our study, the average participant was 43.79 ± 11.96 years old in 2000 and 49.79 ± 11.96 in 2006. Thus, they were probably too young to observe a decline change in the DBP trajectory. Future cohort studies enrolling senior participants and longer observational time are required to elucidate the DBP trajectory in the Chinese population.

By GMM, we identified three classes of trajectories for both SBP and DBP among the non-hypertensive Chinese population, with gender, age and BMI adjusted. Due to the limited epidemiologic evidence focusing on the BP trajectories among non-hypertension individuals, we found some studies reported PB trajectories among the general Chinese population. Fan, J. H. and colleagues studied the association between longitudinal change in BP and risk of mortality in a Chinese county [28]. Instead of using GMM, they defined the BP trajectory patterns by measurements of BP at two-time points (the beginning and the end of observation) [28]. Among the total six patterns, three patterns were similar to us: stable normotension BP, stable prehypertension BP, and prehypertension to hypertension [28]. We did not detect the other three patterns, the normotension to prehypertension, the normotension to hypertension, and prehypertension to normotension. A possible explanation was the difference in the method used to decide BP trajectory patterns. GMM enabled us to identify latent classes of BP trajectories and to estimate the average of BP values in certain BP trajectory (Figure 2 and Figure 3). This was superior to the method applied by Fan, J. H. and colleagues. BP information of every wave was comprehensively considered in our study; by contrast, only two measurements were used in the study conducted by Fan, J. H. and colleagues. In addition, the BP may fluctuate during the observational period, and decreased BP may represent a transient change under the threshold of 120/80 mm Hg that may not reflect the actual pattern of BP change. This was confirmed in our study (data not shown): some individuals had low BP in one certain wave which rebounded in the next wave.

Increased risks of stroke and MI among classes with increasing BP trajectories were observed, The range of aHR was 1.369 to 3.837 of increased SBP trajectories and stroke and aHR was 6.047 to 13.017 of increased SBP trajectories and MI. Li, W. and colleagues discussed the association between long-term SBP patterns in community-dwelling adults and the risk of intracerebral hemorrhage and cerebral infarction [40]. Two trajectories among five trajectories patterns of SBP were similar to our study: the normotensive-stable class and prehypertension-stable class. Statistically significant increased risk of stroke was observed, which was comparable to our study. Compared to the normotensive-stable class, the aHR (95% CI) in the prehypertension-stable class was 3.11 (1.72–5.64) for intracerebral hemorrhage and 1.99 (1.60–2.49) for cerebral infarction [40]. However, Xu, Y. and colleagues conducted a study using the dataset of the CHNS and reported a null association between CVDs and trajectories of SBP [29]. Using LCGM, Xu, Y. and colleagues identified five SPB trajectories, a including rapid increase group, slight increase group, stable group, increase group, and fluctuant group. Except for the fluctuant group, the other four groups were similar to our study: they started under 120 mmHg and later increased at different speeds. Compared to the slight increase group, no statistically significant association of risk of cardiovascular diseases were identified in neither the stable group (adjusted odds ratio, aOR (95% CI): 0.79 (0.40–1.59)) nor the increase group (aOR (95% CI): 1.36 (0.82–2.26)) [29]. We hypothesized that this difference was related to the character of individuals enrolled and statistical methods applied. Hypertension is a well-recognized risk factor for CVDs; however, Xu, Y. and colleagues did not exclude hypertensive individuals at the baseline [29]. The association between BP trajectories and the risk of CVDs may be distorted by the uneven distribution of hypertensive individuals among different classes, which was partly proven by the sensitivity analyses conducted by Xu, Y. and colleagues. When stratified by the use of antihypertensive drugs, compared to the slight increase group, the aOR (95% CI) among the stable group was inversed (aOR (95% CI): 1.37 (0.25–7.52) versus aOR (95% CI): 0.79 (0.40–1.59) without stratification), although the association was still statistically insignificant. In addition, although Xu, Y. and colleagues considered the age in modeling the trajectory of SBP, other covariates that influence the BP were left out [29]. For example, different gender followed a different pattern of BP change [47] and BMI was also related to BP change [50]. By contrast, we adjusted multiple covariates in modeling trajectories, including age, gender, and BMI, which enabled us to better explore the BP trajectories and revealed the relationship between BP trajectories and CVDs. Furthermore, although with a cohort design, Xu, Y. and colleagues used logistic regression to discuss the association of SBP trajectories and the risk of cardiovascular diseases [29]. This may blur the association between CVDs and different trajectories of SBP because the information of survival time was eliminated in the analyses. By contrast, our study divided the observation waves into two stages, the exposure period (wave 2000, 2004, and 2006) and the outcome period (wave 2006, 2009, 2011, and 2015), and applied the Cox regression which considered the combined information of trajectories of BP, survival time of onset of stroke or MI, which can comprehensively reveal the association between CVDs and trajectories of BP.

Previous studies reported that SBP is an independent risk predictor for CVDs among the hypertensive population [51]; our finding also showed a similar result that the trajectories of SBP were more important risk factors than the ones of DBP in stroke and MI among the non-hypertensive population. We used a forward stepwise method in Cox regression models, the association between trajectories of SPB and stroke and MI remained significant after being adjusted for trajectories of DBP while the variable of trajectories of DBP was left out by the models. This indicated that individuals should pay more attention to the management of SBP to prevent stroke and MI.

Our finding of trajectories of BP and the association between patterns of BP and risk of stroke and MI could bring a clue for preventive policy and treatment guidelines in the future. According to the China Hypertension Survey, a stratified multistage study involving 487,349 participants from 31 provinces of mainland China, the prevalence of prehypertension was 41.3% in 2015 [13]. This indicated that about 435.3 million Chinese were at risk of progression to hypertension and faced increased risk of various diseases, including vascular lesion, heart and kidney disorders, and cognitive impairment [13,15,16,17,18,19,20,21]. In the latest Chinese guidelines for the management of hypertension, pre-hypertensive individuals are recommended to receive lifestyle interventions and medical follow-up every 1 to 3 months, while antihypertensive drugs are not recommended due to the lack of clinical studies relating to prognosis [32]. However, several studies have shown the efficiency of antihypertension drugs to reduce the progress of hypertension among pre-hypertensive individuals [52,53,54]. A meta-analysis of 147 randomized trials summarized the use of antihypertensive drugs in the prevention of cardiovascular diseases, which found that a reduction of 10 mm Hg SBP and 5 mm Hg DBP in a population without vascular diseases would effectively decrease the risk of CHD events (relative risk, RR (95% CI): 0.79 (0.72–0.86)) and stroke (RR (95% CI): 0.54 (0.45–0.65)) [22]. In addition, according to the Seventh Report of the Joint National Committee on Prevention, Detection, Evaluation, and Treatment of High Blood Pressure, drugs are recommended when prehypertension individuals show compelling indications, such as chronic kidney disease or diabetes [51]. Further studies should focus on the issue which relates to designing effectiveness, safety, and economical CVD preventive programs and evaluating the feasibility of applying antihypertensive drugs to prevent hypertension and lower CVDs risk among the pre-hypertensive Chinese population.

With a large sample size and a national-level cohort follow-up for 15 years, the statistical power of this study was warranted. By applying GMM with multiple covariates adjusted for, we were able to accurately categorize subgroup trajectories of BP which followed similar growth patterns. However, the study has several limitations. Firstly, because of the study design of the CHNS, information about hypertension, stroke, MI, and some covariates was self-reported; we cannot rule out measurement errors from the interviewers and interviewees. For interviewers, they may mistakenly record the information; this information bias may cause misclassification of the status of diseases. For interviewees, they may have difficulties in recalling information, thus recalling bias exists. In addition, potential self-reporting bias needed to be taken into consideration. Interviewees may be reluctant to answer questions, or they may give the answer that catered to the expectations of others. This may occur in seemingly innocuous information such as age and ethnicity. In the collection process of sensitive information, such as the status of diseases and health-related behaviors, the self-reporting bias was also frequently observed. Secondly, although we tried our best to control confounders and adjusted various factors in our analysis, due to the lack of relevant data, we did not evaluate some other potential confounding factors such as genes, family history, noise exposure, and diet pattern. Previous studies point out that DBP is a more important risk factor in cerebral hemorrhage while SBP is more closely associated with cerebral infarction [55]. However, due to the limited information of the database, we did not differentiate the subtypes of stroke, so we failed to distinguish the association between the subtypes of stroke and SBP and DBP, respectively. Finally, missing values may bias the result. Nonetheless, the effect of missing values might be minimized because the rate was rather low (less than 5%) and the association between BP trajectories and stroke or MI remained unchanged without imputation of missing values (Table S2).

## 5. Conclusions

In summary, our study explored the trajectories of BP and observed a positive association between trajectories of BP and risk of stroke or MI among the non-hypertensive Chinese population. BP management is important among pre-hypertensive adults in order to prevent stroke and MI in their later lives. Well-designed research with a larger sample size is required to confirm our findings and find efficient methods to prevent stroke and MI.

## Figures and Tables

**Table 1:** The characteristics of the participants.

Variables	Dataset of Stroke (*n* = 2877)		Dataset of MI (*n* = 2879)	
	N	%	N	%
**Age group in 2006**				
≤40	640	22.2	642	22.3
41–50	874	30.4	873	30.3
51–60	815	28.3	811	28.2
≥61	548	19.0	553	19.2
**Location**				
Urban	731	25.4	730	25.4
Rural	2146	74.6	2149	74.6
**Ethnic**				
Majority (Han)	2451	85.2	2454	85.2
Minority	426	14.8	425	14.8
**Gender**				
Male	1282	44.6	1283	44.6
Female	1595	55.4	1596	55.4
**Education level**				
Illiteracy	829	28.8	821	28.5
Primary school	595	20.7	603	20.9
Middle school degree	1259	43.8	1257	43.7
Technical or vocational degree and higher	194	6.7	198	6.9
**Smoking in 2006**				
Never	1992	69.2	1994	69.3
Ever	885	30.8	885	30.7
**Still smoking in 2006**				
No	2087	72.5	2090	72.6
Yes	790	27.5	789	27.4
**Drinking in 2006**				
Never	1942	67.5	1942	67.5
Ever	935	32.5	937	32.5
**BMI category in 2006 (kg/m^2^)**				
Underweight	160	5.6	159	5.5
Normal	1681	58.4	1687	58.6
Overweight	818	28.4	816	28.3
Obese	218	7.6	217	7.5
**Energy intake in 2006 ^a^**	2226.01 ± 670.73		2225.37 ± 671.33	
**Activity level in 2006**				
Light	1043	36.3	1043	36.2
Middle	417	14.5	424	14.7
Heavy	1408	48.9	1404	48.8
No working ability	9	0.3	8	0.3

Abbreviation: MI, myocardial infarction. Information in bold in the first rank were names of variables. ^a^ presented by average ± standard deviation.

**Table 2:** The characteristics across blood pressure trajectory groups (systolic pressure and diastolic pressure, respectively) in the dataset of stroke (*N* = 2877).

Variable	Systolic Blood Pressure							Diastolic Blood Pressure						
	Class 1 (*n* = 277)		Class 2 (*n* = 1713)		Class 3 (*n* = 887)		*P*	Class 1 (*n* = 1035)		Class 2 (*n* = 1793)		Class 3 (*n* = 49)		*P*
	N	%	N	%	N	%		N	%	N	%	N	%	
**Event**	15	5.4%	26	1.5%	3	0.3%	<0.001 ^b^	6	0.6%	38	2.1%	0	0.0%	0.004 ^b^
**Age group in 2006**														
≤40	1	0.4%	242	14.1%	397	44.8%	<0.001	380	36.7%	257	14.3%	3	6.1%	<0.001
41–50	29	10.5%	483	28.2%	362	40.8%		443	42.8%	420	23.4%	11	22.4%	
51–60	82	29.6%	610	35.6%	123	13.9%		205	19.8%	595	33.2%	15	30.6%	
≥61	165	59.6%	378	22.1%	5	0.6%		7	0.7%	521	29.1%	20	40.8%	
**Location**														
Urban	59	21.3%	442	25.8%	230	25.9%	0.255	273	26.4%	447	24.9%	11	22.4%	0.620
Rural	218	78.7%	1271	74.2%	657	74.1%		762	73.6%	1346	75.1%	38	77.6%	
**Ethnicity**														
Majority (Han)	240	86.6%	1447	84.5%	764	86.1%	0.409	896	86.6%	1515	84.5%	40	81.6%	0.254
Minority	37	13.4%	266	15.5%	123	13.9%		139	13.4%	278	15.5%	9	18.4%	
**Gender**														
Male	125	45.1%	1094	63.9%	63	7.1%	<0.001	38	3.7%	1209	67.4%	35	71.4%	<0.001
Female	152	54.9%	619	36.1%	824	92.9%		997	96.3%	584	32.6%	14	28.6%	
**Education level**														
Illiteracy	137	49.5%	493	28.8%	199	22.4%	<0.001	253	24.4%	554	30.9%	22	44.9%	0.001 ^b^
Primary school	64	23.1%	353	20.6%	178	20.1%		215	20.8%	370	20.6%	10	20.4%	
Middle school	61	22.0%	749	43.7%	449	50.6%		498	48.1%	745	41.6%	16	32.7%	
Technical or vocational degree and higher	15	5.4%	118	6.9%	61	6.9%		69	6.7%	124	6.9%	1	2.0%	
**Smoking in 2006**														
Never	193	69.7%	963	56.2%	836	94.3%	<0.001	995	96.1%	971	54.2%	26	53.1%	<0.001
Ever	84	30.3%	750	43.8%	51	5.7%		40	3.9%	822	45.8%	23	46.9%	
**Still smoking in 2006**														
No	208	75.1%	1039	60.7%	840	94.7%	<0.001	998	96.4%	1063	59.3%	26	53.1%	<0.001
Yes	69	24.9%	674	39.3%	47	5.3%		37	3.6%	730	40.7%	23	46.9%	
**Drinking in 2006**														
Never	190	68.6%	973	56.8%	779	87.8%	<0.001	928	89.7%	996	55.5%	18	36.7%	<0.001
Ever	87	31.4%	740	43.2%	108	12.2%		107	10.3%	797	44.5%	31	63.3%	
**BMI category in 2006 (kg/m^2^)**														
Underweight	24	8.7%	96	5.6%	40	4.5%	<0.001	47	4.5%	109	6.1%	4	8.2%	0.036 ^b^
Normal	154	55.6%	996	58.1%	531	59.9%		608	58.7%	1047	58.4%	26	53.1%	
Overweight	63	22.7%	510	29.8%	245	27.6%		289	27.9%	518	28.9%	11	22.4%	
Obese	36	13.0%	111	6.5%	71	8.0%		91	8.8%	119	6.6%	8	16.3%	
**Energy intake in 2006 ^a^**	2067.19 ± 686.13		2311.11 ± 684.61		2111.26 ± 610.69		<0.001 ^c^	2110.90 ± 608.47		2291.07 ± 694.00		2277.09 ± 743.05		<0.001 ^c^
**Activity level in 2006**														
Light	135	48.7%	594	34.7%	314	35.4%	<0.001 ^b^	365	35.3%	662	36.9%	16	32.7%	0.405 ^b^
Middle	27	9.7%	253	14.8%	137	15.4%		153	14.8%	260	14.5%	4	8.2%	
Heavy	112	40.4%	861	50.3%	435	49.0%		516	49.9%	863	48.1%	29	59.2%	
No working ability	3	1.1%	5	0.3%	1	0.1%		1	0.1%	8	0.4%	0	0.0%	

Information in bold in the first rank were names of variables. ^a^ presented by average ± standard deviation; ^b^ tested by the Fisher exact method; ^c^ tested by Kruskal–Wallis tests.

**Table 3:** The characteristics across blood pressure trajectory groups (systolic pressure and diastolic pressure, respectively) in the dataset of myocardial infarction (*N* = 2879).

Variable	Systolic Blood Pressure							Diastolic Blood Pressure						
	Class 1 (*n* = 1435)		Class 2 (*n* = 215)		Class 3 (*n* = 1229)		*P*	Class 1 (*n* = 952)		Class 2 (*n* = 1438)		Class 3 (*n* = 498)		*P*
	N	%	N	%	N	%		N	%	N	%	N	%	
**Event**	28	2.0%	9	4.2%	4	0.3%	<0.001 ^b^	25	2.6%	15	1.0%	1	0.2%	0.000 ^b^
**Age group in 2006**														
≤40	128	8.9%	2	0.9%	512	41.7%	<0.001	95	10.0%	217	15.1%	330	67.5%	<0.001
41–50	415	28.9%	26	12.1%	432	35.2%		269	28.3%	449	31.2%	155	31.7%	
51–60	518	36.1%	69	32.1%	224	18.2%		321	33.7%	486	33.8%	4	0.8%	
≥61	374	26.1%	118	54.9%	61	5.0%		267	28.0%	286	19.9%	0	0.0%	
**Location**														
Urban	367	25.6%	49	22.8%	314	25.5%	0.668	270	28.4%	341	23.7%	119	24.3%	0.620
Rural	1068	74.4%	166	77.2%	915	74.5%		682	71.6%	1097	76.3%	370	75.7%	
**Ethnicity**														
Majority (Han)	1237	86.2%	187	87.0%	1030	83.8%	0.167	845	88.8%	1182	82.2%	427	87.3%	<0.001
Minority	198	13.8%	28	13.0%	199	16.2%		107	11.2%	256	17.8%	62	12.7%	
**Gender**														
Male	854	59.5%	95	44.2%	334	27.2%	<0.001	598	62.8%	659	45.8%	26	5.3%	<0.001
Female	581	40.5%	120	55.8%	895	72.8%		354	37.2%	779	54.2%	463	94.7%	
**Education level**														
Illiteracy	426	29.7%	100	46.5%	295	24.0%	<0.001	272	28.6%	481	33.4%	68	13.9%	<0.001
Primary school	328	22.9%	49	22.8%	226	18.4%		205	21.5%	293	20.4%	105	21.5%	
Middle school	588	41.0%	53	24.7%	616	50.1%		405	42.5%	580	40.3%	272	55.6%	
Technical or vocational degree and higher	93	6.5%	13	6.0%	92	7.5%		70	7.4%	84	5.8%	44	9.0%	
**Smoking in 2006**														
Never	848	59.1%	153	71.2%	993	80.8%	<0.001	564	59.2%	957	66.6%	473	96.7%	<0.001
Ever	587	40.9%	62	28.8%	236	19.2%		388	40.8%	481	33.4%	16	3.3%	
**Still smoking in 2006**														
No	920	64.1%	161	74.9%	1009	82.1%	<0.001	617	64.8%	999	69.5%	474	96.9%	<0.001
Yes	515	35.9%	54	25.1%	220	17.9%		335	35.2%	439	30.5%	15	3.1%	
**Drinking in 2006**														
Never	869	60.6%	145	67.4%	928	75.5%	<0.001	549	57.7%	955	66.4%	438	89.6%	<0.001
Ever	566	39.4%	70	32.6%	301	24.5%		403	42.3%	483	33.6%	51	10.4%	
**BMI category in 2006 (kg/m^2^)**														
Underweight	50	3.5%	7	3.3%	102	8.3%	<0.001	4	0.4%	117	8.1%	38	4	<0.001
Normal	738	51.4%	74	34.4%	875	71.2%		329	34.6%	998	69.4%	360	329	
Overweight	511	35.6%	76	35.3%	229	18.6%		433	45.5%	302	21.0%	81	433	
Obese	136	9.5%	58	27.0%	23	1.9%		186	19.5%	21	1.5%	10	186	
**Energy intake in 2006 ^a^**	2290.27 ± 687.03		2060.53 ± 635.23		2178.43 ± 650.28		<0.001	2288.77 ± 689.68		2225.48 ± 672.58		2101.64 ± 612.98		<0.001 ^c^
**Activity level in 2006**														
Light	544	37.9%	110	51.2%	389	31.7%	<0.001 ^b^	420	44.1%	463	32.2%	160	32.7%	<0.001 ^b^
Middle	207	14.4%	20	9.3%	197	16.0%		136	14.3%	201	14.0%	87	17.8%	
Heavy	678	47.2%	84	39.1%	642	52.2%		394	41.4%	769	53.5%	241	49.3%	
No working ability	6	0.4%	1	0.5%	1	0.1%		2	0.2%	5	0.3%	1	0.2%	

Information in bold in the first rank were names of variables. ^a^ presented by average ± standard deviation; ^b^ tested by the Fisher exact method; ^c^ tested by Kruskal–Wallis tests.

**Table 4:** The associations between blood pressure trajectories and stroke or myocardial infarction (MI) by cox regressions.

Outcomes		Classes of Trajectories	Event	*n*	Incidence Density ^a^	Crude Model			Adjusted Model 1 ^b^			Adjusted Model 2 ^c^		
						HR	95% CI	*p*	HR	95% CI	*p*	HR	95% CI	*p*
Stroke	SBP	Class 3	3	887	0.063	Ref.			Ref.			Ref.		
		Class 1	15	277	1.015	16.400	4.748–56.648	<0.001	3.837	0.911–16.167	0.067	3.837	0.911–16.167	0.067
		Class 2	26	1713	0.282	4.513	1.366–14.910	0.013	1.369	0.357–5.248	0.647	1.369	0.357–5.248	0.647
	DBP	Class 1	6	1035	0.107	Ref.			-	-	-	-	-	-
		Class 2	38	1793	0.395	3.685	1.558–8.718	0.003	-	-	-	-	-	-
		Class 3	0	49	0	-	-	-	-	-	-	-	-	-
MI	SBP	Class 3	4	1229	0.060	Ref.			Ref.			Ref.		
		Class 1	28	1435	0.362	6.047	2.121–17.239	0.001	6.047	2.121–17.239	0.001	6.047	2.121–17.239	0.001
		Class 2	9	215	0.778	13.017	4.009–42.470	<0.001	13.017	4.009–42.270	<0.001	13.017	4.009–42.270	<0.001
	DBP	Class 3	1	498	0.038	Ref.			Ref.			-	-	-
		Class 1	25	952	0.488	12.996	1.761–95.913	0.012	2.821	0.323–24.665	0.349	-	-	-
		Class 2	15	1438	0.193	5.118	0.676–38.742	0.114	1.312	0.150–11.494	0.806	-	-	-

Abbreviation: MI, myocardial infarction; SBP, systolic blood pressure; DBP, diastolic blood pressure; HR, hazard ratio; 95% CI, 95% confidence interval. ^a^ Provided as event/1000 person-years. ^b^ The model was adjusted for age level in 2006, location, ethnicity, gender, education level, smoking in 2006, current smoking in 2006, drinking in 2006, BMI category in 2006, energy intake in 2006, and activity level in 2006. Variables were selected by the forward method (likelihood ratio). ^c^ The model was adjusted for age level in 2006, location, ethnicity, gender, education level, smoking in 2006, current smoking in 2006, drinking in 2006, BMI category in 2006, energy intake in 2006, and activity level in 2006, and classification of diastolic blood pressure trajectories. Variables were selected by the forward method (likelihood ratio).

## Data Availability

Restrictions apply to the availability of these data. Data was obtained from the China Health and Nutrition Survey (CHNS) and are available at https://www.cpc.unc.edu/projects/china/data/datasets (accessed on 21 October 2020) with the permission of CHNS.

## Supplementary Materials

The following are available online at https://www.mdpi.com/1660-4601/18/6/2909/s1, Table S1: Fit indices for growth mixture models for blood pressure and stroke and MI among Chinese adults, Table S2: The associations between blood pressure trajectories and stroke or MI by cox regressions eliminating participants without missing values, Table S3: The associations between 2-class and 4-class blood pressure trajectories and stroke or MI by cox regressions, Table S4: The associations between the age-stratified blood pressure trajectories and stroke or MI by cox regressions, Figure S1: The blood pressure trajectories in the 2-class and 4-class models of systolic blood pressure by GMM for stroke, Figure S2: The blood pressure trajectories in the 2-class and 4-class models of diastolic blood pressure by GMM for stroke, Figure S3: The blood pressure trajectories in the 2-class and 4-class models of systolic blood pressure by GMM for myocardial infarction, Figure S4: The blood pressure trajectories in the 2-class and 4-class models of diastolic blood pressure by GMM for myocardial infarction, Figure S5: The blood pressure trajectories in the 3-class model of systolic blood pressure by GMM for stroke, age-stratified, Figure S6, The blood pressure trajectories in the 3-class model of diastolic blood pressure by GMM for stroke, age-stratified, Figure S7: The blood pressure trajectories in the 3-class model of systolic blood pressure by GMM for myocardial infarction, age-stratified, Figure S8: The blood pressure trajectories in the 3-class model of diastolic blood pressure by GMM for MI, age-stratified.
