# Supplementary material for: The Association between Blood Pressure Trajectories and Risk of Cardiovascular Diseases among Non-Hypertensive Chinese Population: A Population-Based Cohort Study
Source: Int J Environ Res Public Health. 2021 Mar 12;18(6):2909. doi: 10.3390/ijerph18062909 (PMC7999224; doi:10.3390/ijerph18062909)
Supplement: Supplementary file 1 [file ijerph-18-02909-s001.pdf]

Table S1. Fit indices for growth mixture models for blood pressure and stroke and MI among Chinese adults.

Outcomes	Variable	Classes	K	G^2/LL	aBIC	Entropy	aLMR	BLRT	Classification Probability	Class Counts
Stroke	SBP	2	16	−34602.882	69282.359	0.716	<0.001	<0.001	2360/517	0.8203/0.1797
		**3**	**21**	**−34489.097**	**69078.724**	**0.641**	**0.0845**	**<0.001**	**277/1713/887**	**0.09628/0.59541/0.30831**
		4	26	−34490.281	69105.028	0.713	0.6711	1	1605/147/0/1125	0.55787/0.05109/0/0.39103
	DBP	2	16	-31364.344	62805.282	0.541	<0.001	<0.001	1767/1110	0.61418/0.38582
		**3**	**21**	**−31327.552**	**62755.633**	**0.666**	**0.0032**	**<0.001**	**1793/1035/49**	**0.62322/0.35975/0.01703**
		4	26	−31327.552	62779.569	0.735	0.4997	0.9934	1035/0/1793/49	0.35975/0/0.62322/0.01703
MI	SBP	2	14	−34779.728	69626.486	0.672	<0.001	<0.001	0.74679/0.25321	2150/729
		**3**	**20**	**−34631.418**	**69358.593**	**0.663**	**0.0114**	**<0.001**	**0.49844/0.07468/0.42688**	**1435/215/1229**
		4	26	−34616.125	69356.735	0.733	0.1583	<0.001	0.01077/0.47378/0.4446/0.07086	31/1364/1280/204
		5	32	−34631.418	69416.047	0.77	0.2398	1.000	0.07468/0.49844/0.42688/0/0	215/1435/1229/0/0
	DBP	2	14	−31469.568	63006.165	0.577	<0.001	<0.001	0.59917/0.40083	1725/1154
		**3**	**20**	**−31436.085**	**62967.927**	**0.7**	**0.0512**	**<0.001**	**0.33067/0.16985/0.49948**	**952/489/1438**
		4	26	−31406.794	62938.073	0.732	0.1965	<0.001	0.1563/0.01146/0.37721/0.45502	450/33/1086/1310
		5	32	−31436.085	63025.381	0.795	0.5513	1	0/0.16985/0.33067/0/0.49948	0/489/952/0/1438

The best-fitting models were presented in bold. Abbreviation: MI, myocardial infarction; SBP, systolic blood pressure; DBP, diastolic blood pressure; G^2/LL, likelihood ratio chi-square; aBIC, sample size-adjusted Bayesian Information Criterion; aLMR, adjusted Lo–Mendell–Rubin likelihood ratio test; BLRT, bootstrapped likelihood ratio test.

Table S2. The associations between blood pressure trajectories and stroke or MI by cox regressions eliminating participants without missing values.

Outcomes		Classes of Trajectories	Event	*n*	Incidence Density [a]	Crude Model			Adjusted Model 1 [b]			Adjusted Model 2 [c]		
						HR	95% CI	*P*	HR	95% CI	*P*	HR	95% CI	*P*
Stroke	SBP	Class 3	3	887	0.063	Ref.			Ref.			Ref.		
		Class 1	15	277	1.015	16.400	4.748–56.648	<0.001	4.513	1.120–18.187	0.034	4.513	1.120–18.187	0.034
		Class 2	26	1713	0.282	4.513	1.366–14.910	0.013	1.680	0.462–6.114	0.431	1.680	0.462–6.114	0.431
	DBP	Class 1	6	1035	0.107	Ref.			-			-	-	-
		Class 2	38	1793	0.395	3.685	1.558–8.718	0.003	-	-	-	-	-	-
		Class 3	0	49	0	-	-	-	-	-	-	-	-	-
MI	SBP	Class 3	4	1229	0.060	Ref.			Ref.			Ref.		
		Class 1	28	1435	0.362	5.841	2.044–16.693	0.001	5.841	2.044–16.693	0.001	5.841	2.044–16.693	0.001
		Class 2	9	215	0.778	13.385	4.122–43.463	0.000	13.320	4.102–43.252	<0.001	13.320	4.102–43.252	<0.001
	DBP	Class 3	1	498	0.038	Ref.			-			-	-	-
		Class 1	25	952	0.488	12.996	1.761–95.913	0.012	-	-	-	-	-	-
		Class 2	15	1438	0.193	5.118	0.676–38.742	0.114	-	-	-	-	-	-

Abbreviation: MI, myocardial infarction; SBP, systolic blood pressure; DBP, diastolic blood pressure; HR, hazard ratio; 95% CI, 95% confidence interval. [a] Provided as event/1000 person-years. [b] The model was adjusted for age level in 2006, location, ethnicity, gender, education level, smoking in 2006, current smoking in 2006, drinking in 2006, BMI category in 2006, energy intake in 2006, and activity level in 2006. Variables were selected by the forward method (likelihood ratio). [c] The model was adjusted for age level in 2006, location, ethnicity, gender, education level, smoking in 2006, current smoking in 2006, drinking in 2006, BMI category in 2006, energy intake in 2006, and activity level in 2006, and classification of diastolic blood pressure trajectories. Variables were selected by the forward method (likelihood ratio).

Table S3. The associations between 2-class and 4-class blood pressure trajectories and stroke or MI by cox regressions.

Outcomes		Class Type	Classes of Trajectories	Event	*n*	Incidence Density [a]	Crude Model			Adjusted Model 1 [b]			Adjusted Model 2 [c]		
							HR	95% CI	*P*	HR	95% CI	*P*	HR	95% CI	*P*
Stroke	SBP	2 class	Class 1	19	2360	0.897	Ref.			Ref.			Ref.		
			Class 2	25	517	5.425	6.113	3.367–11.101	<0.001	3.495	1.729–7.064	<0.001	3.596	1.777–7.277	<0.001
		4 class [d]	Class 1	10	1605	0.693	Ref.			Ref.			Ref.		
			Class 2	8	147	6.135	8.965	3.538–22.716	<0.001	2.692	0.905–8.012	0.075	2.692	0.905–8.012	0.075
			Class 4	26	1125	2.582	3.739	1.803–7.753	<0.001	1.044	0.412–2.643	0.927	1.044	0.412–2.643	0.927
	DBP	2-class	Class 2	6	1110	0.601	Ref.			-	-	-	-	-	-
			Class 1	38	1767	2.403	4.012	1.696–9.490	0.002	-	-	-	-	-	-
		4-class [e]	Class 1	6	1035	0.645	Ref.			-	-	-	-	-	-
			Class 3	38	1793	2.368	3.685	1.558–8.718	0.003	-	-	-	-	-	-
			Class 4	0	49	0.000	-	-	-	-	-	-	-	-	-
MI	SBP	2-class	Class 1	21	2150	1.086	Ref.			-	-	-	-	-	-
			Class 2	20	729	3.063	2.837	1.538–5.234	0.001	-	-	-	-	-	-
		4-class	Class 3	6	1280	0.521	Ref.			-	-	-	-	-	-
			Class 1	0	31	0.000	-	-	-	-	-	-	-	-	-
			Class 2	27	1364	2.206	4.261	1.759–10.321-	0.001	-	-	-	-	-	-
			Class 4	8	204	4.374	8.473	2.940–24.420	<0.001	-	-	-	-	-	-
	DBP	2-class	Class 1	15	1725	0.967	Ref.			-	-	-	-	-	-
			Class 2	26	1154	2.513	2.613	1.384–4.933	0.003	-	-	-	-	-	-
		4-calss	Class 1	1	450	0.247	Ref.			-	-	-	-	-	-
			Class 2	2	33	6.780	27.914	2.531–307.84	0.007	-	-	-	-	-	-
			Class 3	24	1086	2.465	10.051	1.360–74.296	0.024	-	-	-	-	-	-
			Class 4	14	1310	1.188	4.823	0.634–36.679	0.128	-	-	-	-	-	-

Abbreviation: MI, myocardial infarction; SBP, systolic blood pressure; DBP, diastolic blood pressure; HR, hazard ratio; 95% CI, 95% confidence interval. [a] Provided as event/1000 person-years. [b] The model was adjusted for age level in 2006, location, ethnicity, gender, education level, smoking in 2006, current smoking in 2006, drinking in 2006, BMI category in 2006, energy intake in 2006, and activity level in 2006. Variables were selected by the forward method (likelihood ratio). [c] The model was adjusted for age level in 2006, location, ethnicity, gender, education level, smoking in 2006, current smoking in 2006, drinking in 2006, BMI category in 2006, energy intake in 2006, and activity level at 2006, and corresponding classification of diastolic blood pressure trajectories. Variables were selected by the forward method (likelihood ratio). [d] The count of Class 3 was zero. [e] The count of Class 2 was zero.

Table S4. The associations between the age-stratified blood pressure trajectories and stroke or MI by cox regressions.

Outcomes	Age		Classes of Trajectories	Event	*n*	Incidence Density [a]	Crude Model			Adjusted Model 1 [b]			Adjusted Model 2 [c]		
							**HR**	**95% CI**	***P***	**HR**	**95% CI**	***P***	**HR**	**95% CI**	***P***
Stroke	≤50	SBP	Class 3	0	31	0	-	-	-	-	-	-	-	-	-
			Class 1	2	934	0.238	Ref.			-	-	-	-	-	-
			Class 2	5	549	1.016	4.265	0.827–21.982	0.083	-	-	-	-	-	-
	>50		Class 3	14	531	2.948	Ref.			-	-	-	-	-	-
			Class 1	2	67	3.328	1.133	0.258–4.985	0.869	-	-	-	-	-	-
			Class 2	21	765	3.068	1.040	0.529–2.046	0.909	-	-	-	-	-	-
	≤50	DBP	Class 3	1	618	0.180	Ref.			Ref.			Ref.		
			Class 1	2	135	1.667	18.486	2.066–165.394	0.009	18.486	2.066–165.394	0.009	18.486	2.066–165.394	0.009
			Class 2	4	761	0.585	1.624	0.147–17.908	0.692	1.624	0.147–17.908	0.692	1.624	0.147–17.908	0.692
	>50		Class 3	10	537	2.079	Ref.			-	-	-	-	-	-
			Class 1	8	312	2.865	1.380	0.545–3.498	0.497	-	-	-	-	-	-
			Class 2	19	514	4.138	2.001	0.931–4.304	0.076	-	-	-	-	-	-
MI	≤50	SBP	Class 3	1	631	0.176	Ref.			Ref.			Ref.		
			Class 1	3	131	2.549	45.743	4.758–439.751	0.001	44.305	4.607–426.110	0.001	44.305	4.607–426.110	0.001
			Class 2	5	753	0.738	4.833	0.565–41.367	0.150	5.154	0.601–44.202	0.135	5.154	0.601–44.202	0.135
	>50		Class 2	14	789	1.975	Ref.			-	-	-	-	-	-
			Class 1	2	68	3.284	1.679	0.381–7.385	0.493	-	-	-	-	-	-
			Class 3	16	507	3.525	1.792	0.875–3.671	0.111	-	-	-	-	-	-
	≤50	DBP	Class 3	1	720	0.154	Ref.			Ref.			-	-	-
			Class 1	3	48	6.977	14.537	1.512–139.750	0.020	14.340	1.492–137.873	0.021	-	-	-
			Class 2	5	747	0.744	4.202	0.491–35.964	0.190	4.480	0.522–38.418	0.171	-	-	-
	>50		Class 1	7	418	1.867	Ref.			-	-	-	-	-	-
			Class 2	17	567	3.348	1.796	0.745–4.330	0.192	-	-	-	-	-	-
			Class 3	8	379	2.347	1.253	0.454–3.455	0.663	-	-	-	-	-	-

Abbreviation: MI, myocardial infarction; SBP, systolic blood pressure; DBP, diastolic blood pressure; HR, hazard ratio; 95% CI, 95% confidence interval. [a] Provided as event/1000 person-years. [b] The model was adjusted for age level in 2006, location, ethnicity, gender, education level, smoking in 2006, current smoking in 2006, drinking in 2006, BMI category in 2006, energy intake in 2006, and activity level in 2006. Variables were selected by the forward method (likelihood ratio). [c] The model was adjusted for age level in 2006, location, ethnicity, gender, education level, smoking in 2006, current smoking in 2006, drinking in 2006, BMI category in 2006, energy intake in 2006, and activity level in 2006, and corresponding classification of diastolic blood pressure trajectories. Variables were selected by the forward method (likelihood ratio).

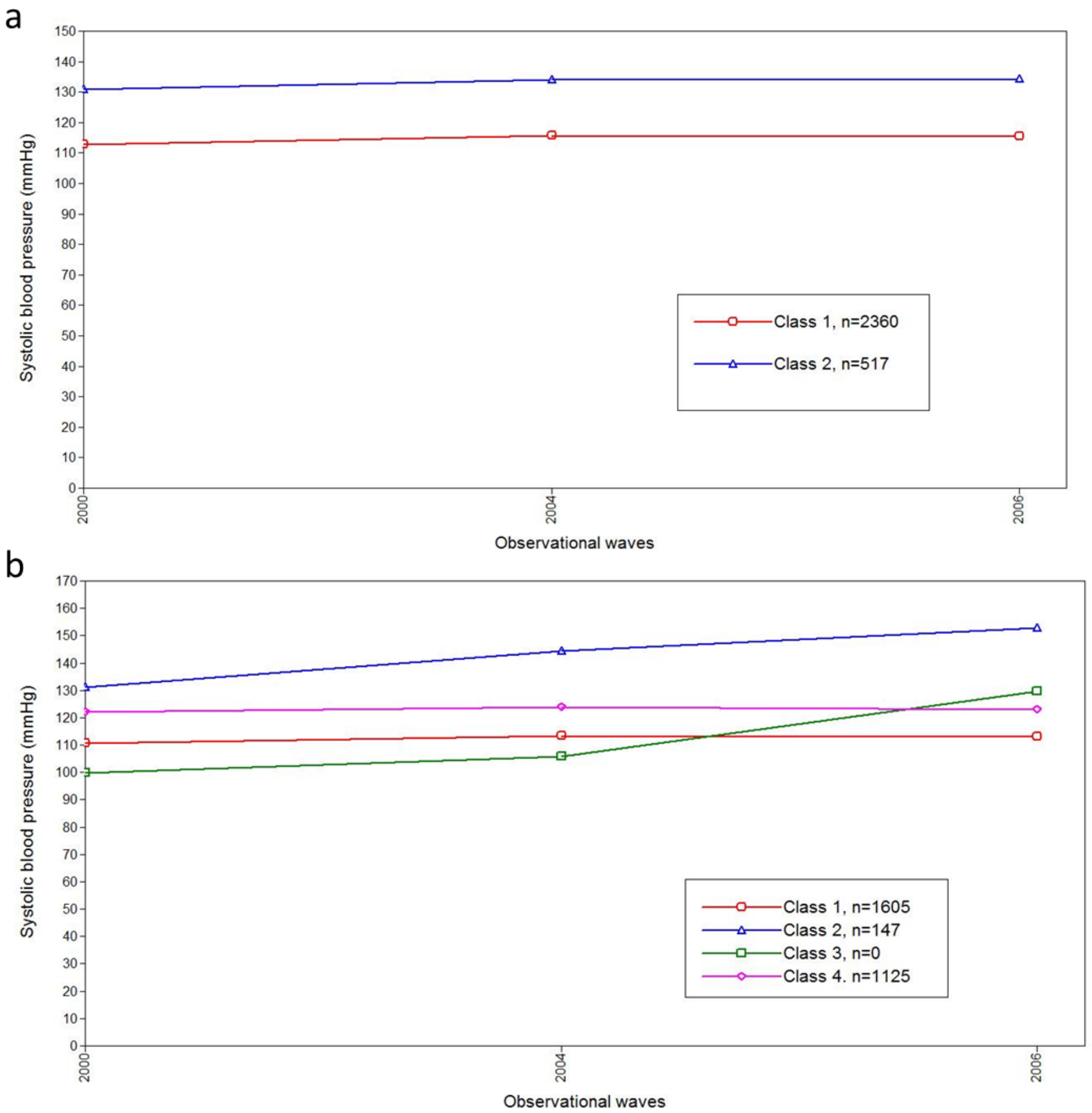

Figure S1. The blood pressure trajectories in the 2-class and 4-class models of systolic blood pressure by GMM for stroke. (**a**) The plots of 2-class systolic blood pressure trajectories by GMM for stroke. (**b**) The plots of 4-class systolic blood pressure trajectories by GMM for stroke. Abbreviations: mmHg, millimeter of mercury; GMM, growth mixture model.

a

b

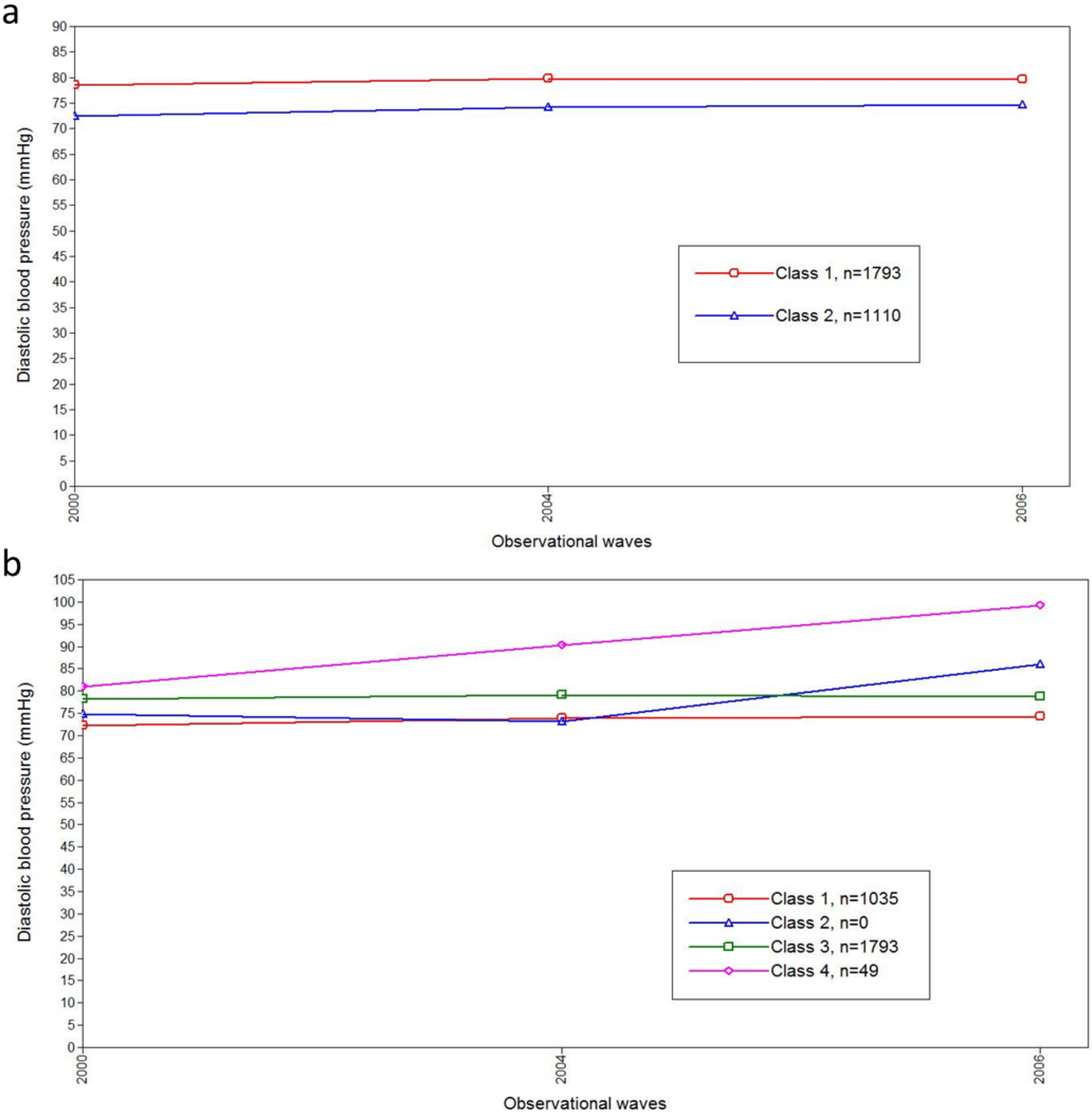

Figure S2. The blood pressure trajectories in the 2-class and 4-class models of diastolic blood pressure by GMM for stroke. (**a**) The plots of 2-class diastolic blood pressure trajectories by GMM for stroke. (**b**) The plots of 4-class diastolic blood pressure trajectories by GMM for stroke. Abbreviations: mmHg, millimeter of mercury; GMM, growth mixture model.

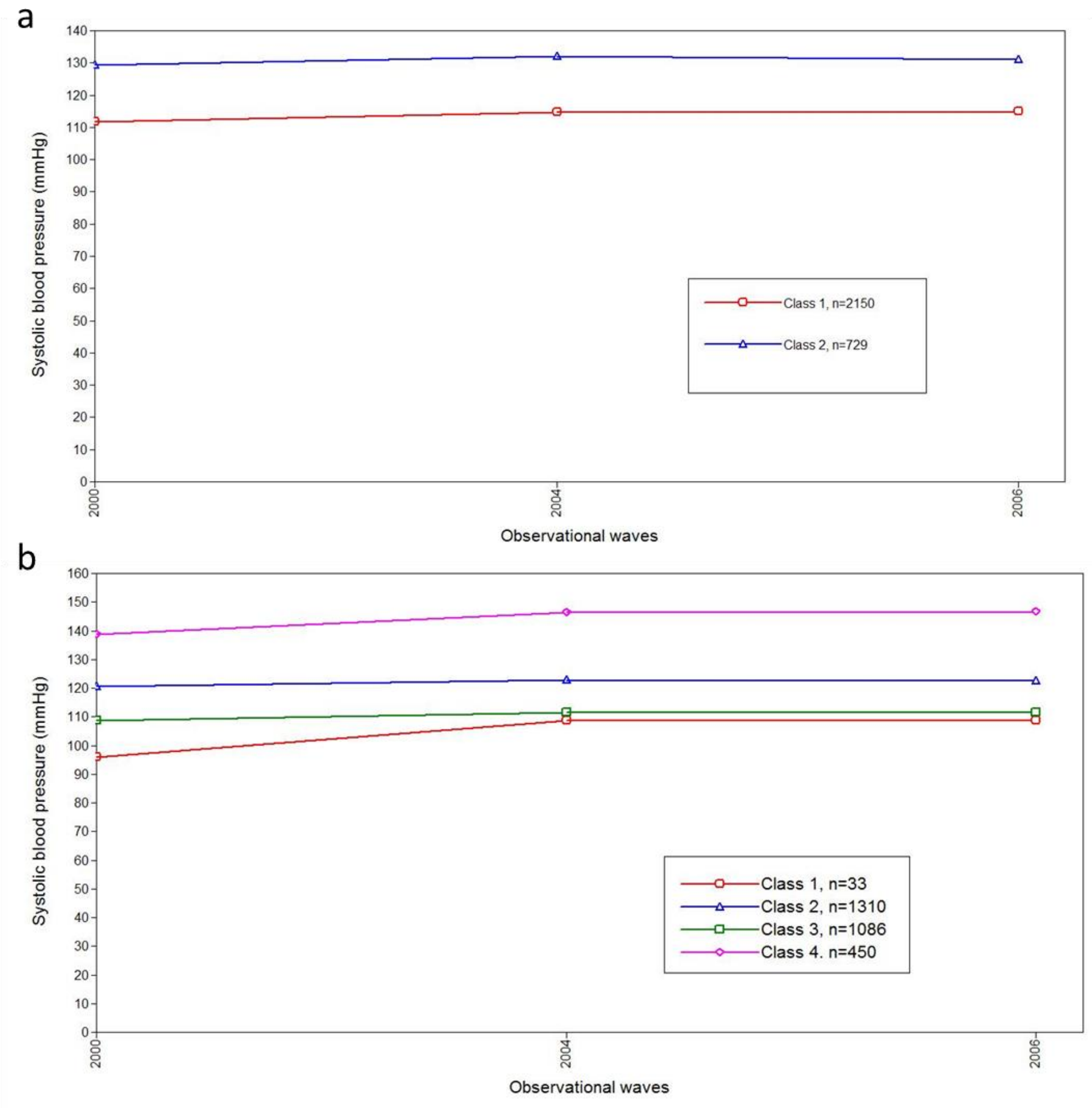

.

Figure S3. The blood pressure trajectories in the 2-class and 4-class models of systolic blood pressure by GMM for myocardial infarction. (**a**) The plots of 2-class systolic blood pressure trajectories by GMM for myocardial infarction. (**b**) The plots of 4-class systolic blood pressure trajectories by GMM for myocardial infarction. Abbreviations: mmHg, millimeter of mercury; GMM, growth mixture model.

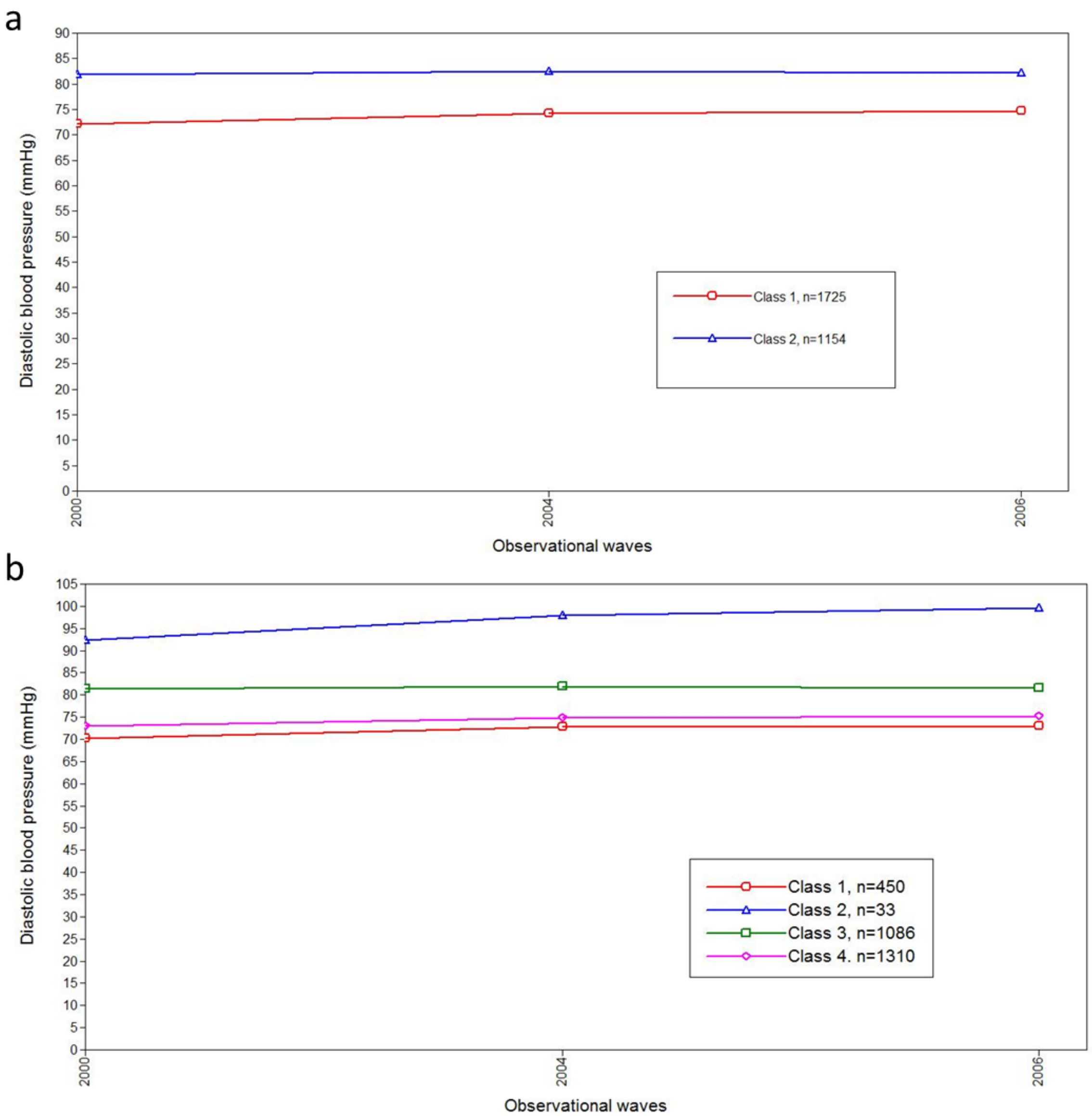

Figure S4. The blood pressure trajectories in the 2-class and 4-class models of diastolic blood pressure by GMM for myocardial infarction. (**a**) The plots of 2-class diastolic blood pressure trajectories by GMM for myocardial infarction. (**b**) The plots of 4-class diastolic blood pressure trajectories by GMM for myocardial infarction. Abbreviations: mmHg, millimeter of mercury; GMM, growth mixture model.

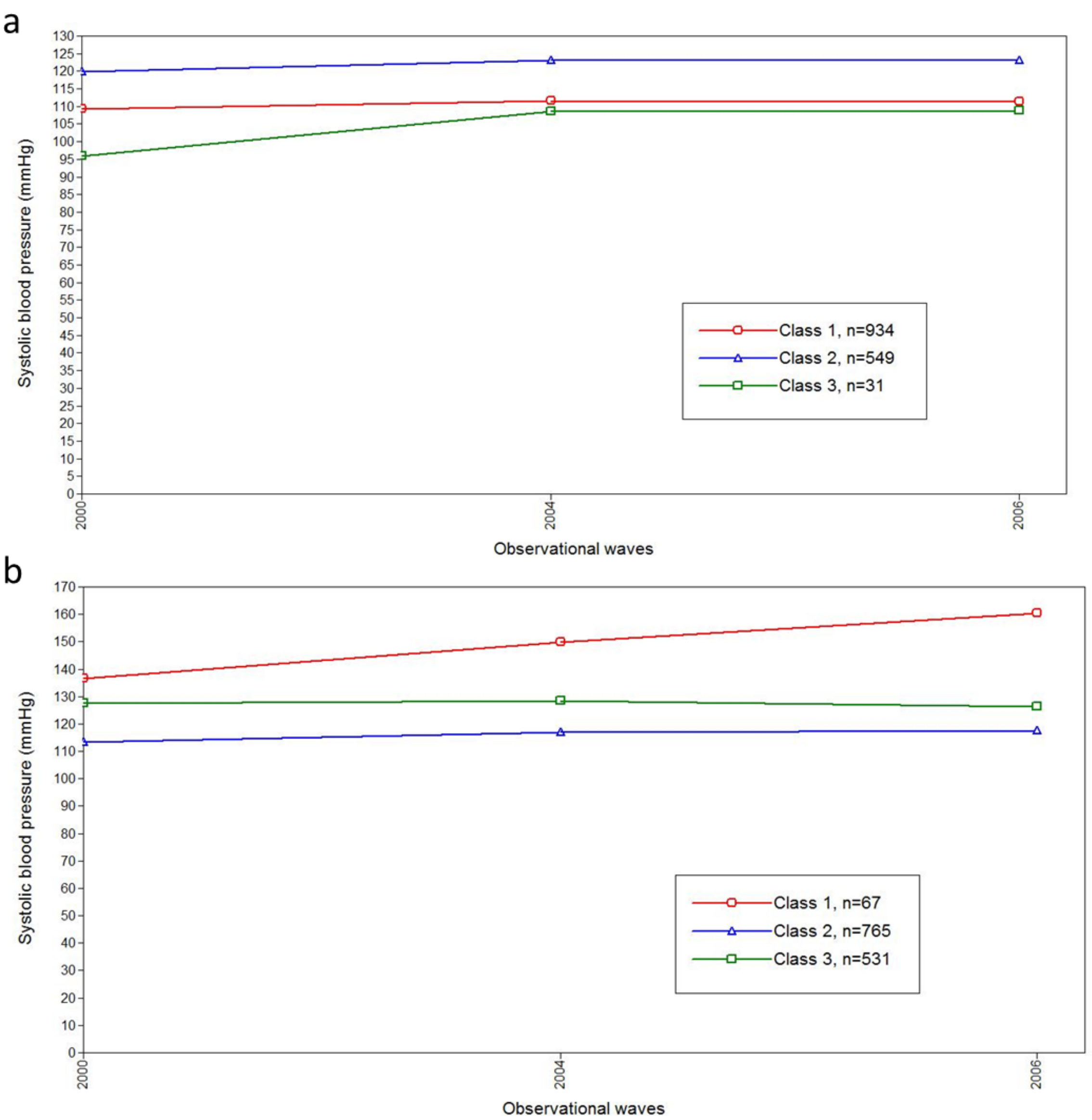

Figure S5. The blood pressure trajectories in the 3-class model of systolic blood pressure by GMM for stroke, age-stratified. (**a**) The plots of 3-class systolic blood pressure trajectories by GMM for stroke, aged 50 and below. (**b**) The plots of 3-class systolic blood pressure trajectories by GMM for stroke, aged above 50. Abbreviations: mmHg, millimeter of mercury; GMM, growth mixture model.

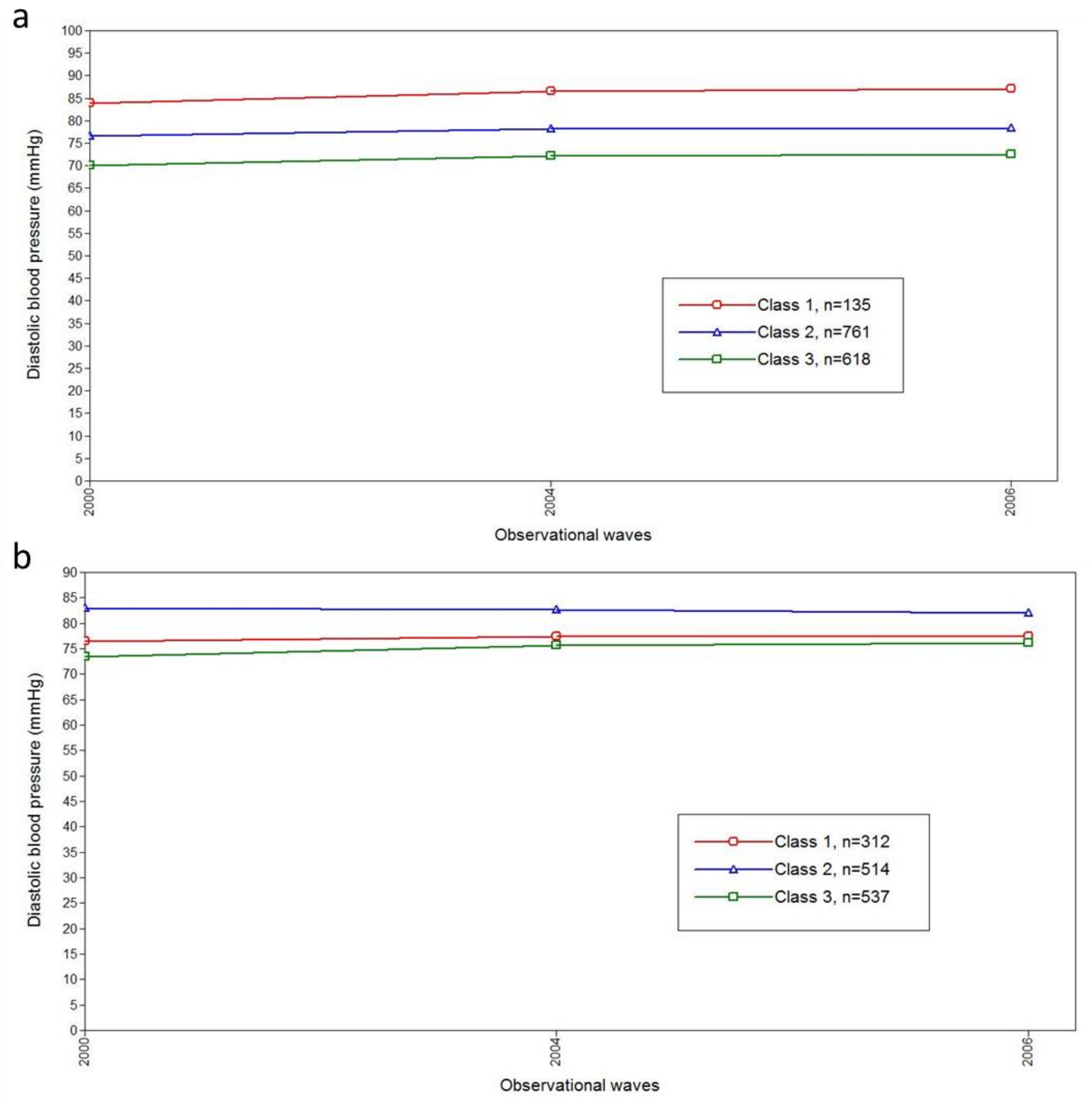

Figure S6. The blood pressure trajectories in the 3-class model of diastolic blood pressure by GMM for stroke, age-stratified. (**a**) The plots of 3-class diastolic blood pressure trajectories by GMM for stroke, aged 50 and below. (**b**) The plots of 3-class diastolic blood pressure trajectories by GMM for stroke, aged above 50. Abbreviations: mmHg, millimeter of mercury; GMM, growth mixture model.

a

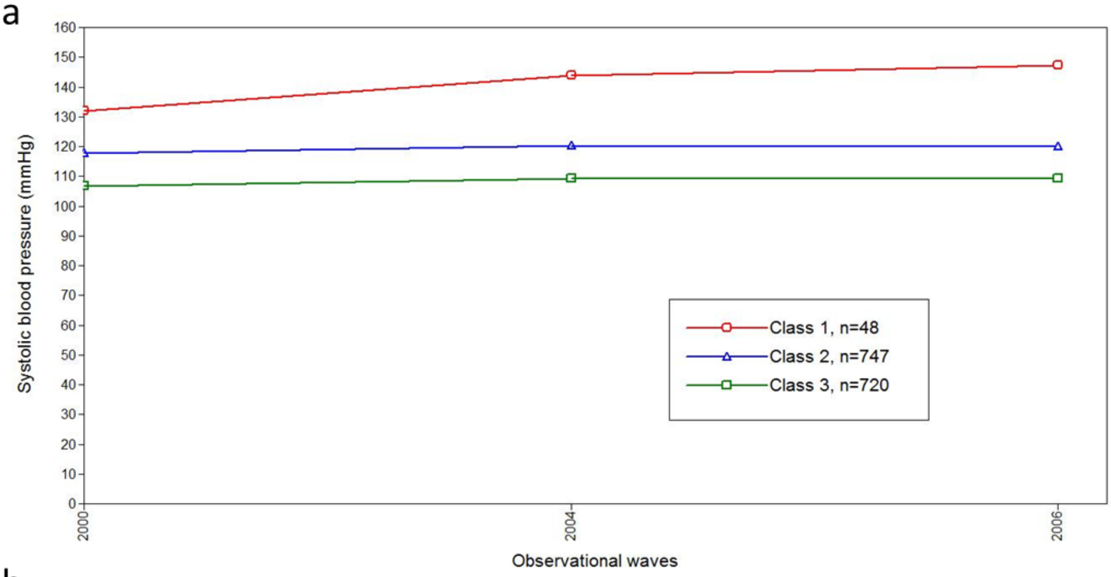

b

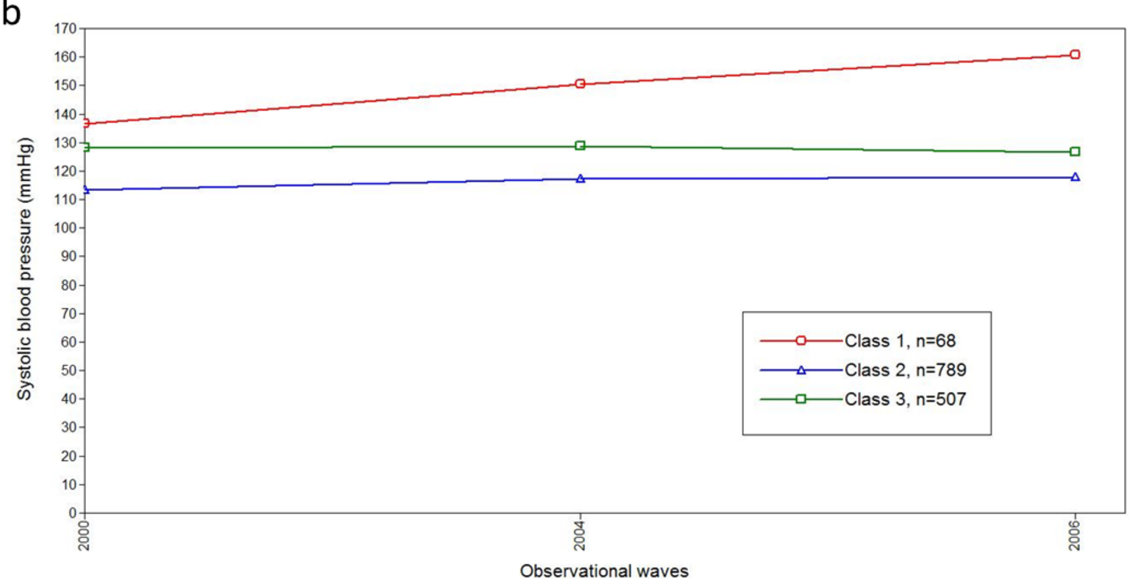

Figure S7. The blood pressure trajectories in the 3-class model of systolic blood pressure by GMM for myocardial infarction, age-stratified. (**a**) The plots of 3-class systolic blood pressure trajectories by GMM for myocardial infarction, aged 50 and below. (**b**) The plots of 3-class systolic blood pressure trajectories by GMM for myocardial infarction, aged above 50. Abbreviations: mmHg, millimeter of mercury; GMM, growth mixture model.

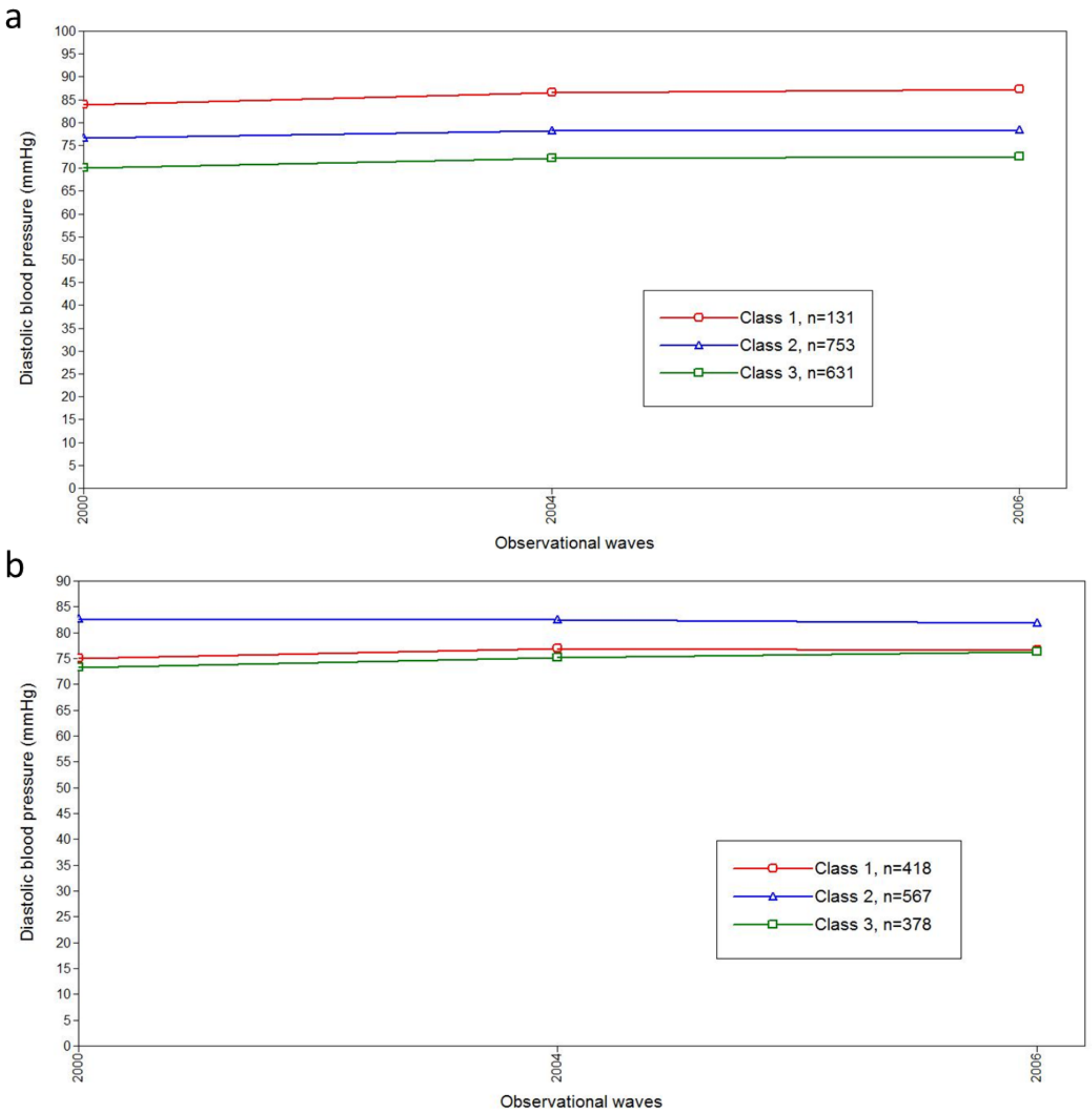

Figure S8. The blood pressure trajectories in the 3-class model of diastolic blood pressure by GMM for MI, age-stratified. (**a**) The plots of 3-class diastolic blood pressure trajectories by GMM for myocardial infarction, aged 50 and below. (**b**) The plots of 3-class diastolic blood pressure trajectories by GMM for myocardial infarction, aged above 50. Abbreviations: mmHg, millimeter of mercury; GMM, growth mixture model.
